# Phantom study of a fully automatic radioactive seed placement robot for the treatment of skull base tumours

**DOI:** 10.1186/s12903-024-04089-w

**Published:** 2024-04-05

**Authors:** Meng Fanhao, Xie Dongsheng, Jin Nenghao, Song Yu, Tian Huanyu, Qiao Bo, Liang Bofu, Zhang Ning, Chang Shimin, Gao Runtao, Duan Xingguang, Zhang Haizhong

**Affiliations:** 1 Department of Oral and Maxillofacial Surgery, Beijing Friendship Hospital, Capital Medical University, Beijing, China; 2 School of Medical Technology, Beijing Institute of Technology, Beijing, China; 3 Department of Stomatology, The First Medical Centre, Chinese PLA General Hospital, Beijing, China; 4 Department of Prosthodontics, Beijing Citident Stomatology Hospital, Beijing, China; 5 School of Mechatronical Engineering, Beijing Institute of Technology, Beijing, China; 6 Clinical Engineering Department, Beijing Baihui Weikang Technology Co., Ltd, Beijing, China

**Keywords:** Automatic radioactive seed placement, Robotic-assisted surgery, Brachytherapy, Skull base tumour, Automated needle insertion, Remebot

## Abstract

**Background:**

Interstitial brachytherapy is a form of intensive local irradiation that facilitates the effective protection of surrounding structures and the preservation of organ functions, resulting in a favourable therapeutic response. As surgical robots can perform needle placement with a high level of accuracy, our team developed a fully automatic radioactive seed placement robot, and this study aimed to evaluate the accuracy and feasibility of fully automatic radioactive seed placement for the treatment of tumours in the skull base.

**Methods:**

A fully automatic radioactive seed placement robot was established, and 4 phantoms of skull base tumours were built for experimental validation. All the phantoms were subjected to computed tomography (CT) scans. Then, the CT data were imported into the Remebot software to design the preoperative seed placement plan. After the phantoms were fixed in place, navigation registration of the Remebot was carried out, and the automatic seed placement device was controlled to complete the needle insertion and particle placement operations. After all of the seeds were implanted in the 4 phantoms, postoperative image scanning was performed, and the results were verified via image fusion.

**Results:**

A total of 120 seeds were implanted in 4 phantoms. The average error of seed placement was (2.51 ± 1.44) mm.

**Conclusion:**

This study presents an innovative, fully automated radioactive particle implantation system utilizing the Remebot device, which can successfully complete automated localization, needle insertion, and radioactive particle implantation procedures for skull base tumours. The phantom experiments showed the robotic system to be reliable, stable, efficient and safe. However, further research on the needle-soft tissue interaction and deformation mechanism of needle puncture is still needed.

## Introduction

Brachytherapy is defined as the short-distance treatment of malignant tumours with a radioactive isotope placed on, in, or near the lesion to continuously emit γ-rays [1], perpetually destroying tumour cells [2]. Because the dose decreases exponentially with increasing distance from the radioactive source, tumours can be effectively damaged while minimizing damage to the surrounding normal tissue [3]. Brachytherapy has been widely used in treating several types of cancer, including head and neck [4], prostate [5] and lung [6] cancer. Given the complexity of the anatomical structures in the skull base, which include the eyeball, numerous blood vessels (such as the internal carotid and jugular veins), cranial nerves IX-XII, and spinal cord tissues, all of which are within a confined space [7], resection of head and neck malignancies usually causes facial defects and aesthetic deformities. Additionally, free or pedicled flaps are even sometimes needed for postoperative reconstruction [8, 9]. Brachytherapy has a unique advantage for treating malignant tumours located in the skull base, especially for patients with advanced or recurrent cancer or as a supplementary therapy after surgical resection. However, there is an inevitable issue that persists: staff exposure. Although a single radioactive particle emits minimal radiation doses and has a limited range, the cumulative dose in the target area is relatively high because of the accumulation of radioactive particles in the patient’s body, occasionally reaching levels as high as 120 Gy. Even with the use of protective gear, including lead aprons, collars, and caps, the crystalline lens of the eye remains exposed to radiation during the procedure and is particularly vulnerable to radiation damage.

Currently, the use of robots and advanced technology in brachytherapy is increasing, and there is a substantial body of literature on robot-assisted brachytherapy for lung [10], bladder [11, 12], and prostate [13] cancer. Robot-assisted needle insertion technology will help surgeons perform high-accuracy seed implantation, improve the calculation of optimal seed placement and reduce radiation exposure to medical staff [14]. Moreover, similar to remote after-loading technology, physicians only need to stay in a dedicated shielded room with audio/visual surveillance. Considering the issues described above, the development of an automated radioactive particle implantation device could facilitate the replacement of manual implantation by surgeons via robotic procedures.

Our preliminary experiments suggest that the accuracy of Remebot puncture at the skull base meets clinical requirements [15]. The robotic arm moves axially with precision, as directed by the surgeon, enabling precise linear movement towards or away from the target point, potentially allowing robot to replace surgeons in conducting puncture and injection procedures. This study aimed to assess the accuracy and feasibility of a fully automated radioactive seed implantation robot for treating skull base tumours.

## Materials and methods

Simulation skull models (1:1, PNATOMY, Zhangjiagang Kexin Scientific Instrument Co., Ltd., Suzhou, China), ultralight clay (Ninitegong, Shandong Zhiqi Hangyi Puzzle Toy Co., Ltd., Linyi, China), a large amount of Kirschner wire (diameter 6 mm, length 200 mm), an appropriate amount of fresh pork and pigskin, MK06A markers (Beijing Baihuiweikang Technology Co., Ltd., Beijing, China), a Remebot robot, and 3 − 0 sutures were used.

### Fully automatic radioactive seed placement robot

The end-effector, a fully automatic radioactive seed placement device consisting mainly of a quick-release connector, linear module, calibration plate and radioactive seed cartridge, was rigidly mounted on the Remebot flange. The linear module consisted of three parts: a stepping motor, an actuator and a ball screw. The ball screw mechanism could convert the rotating motion of the motor into linear motion by controlling the forwards and reverse motion of the stepper motor to realize the insertion and withdrawal of the needle. The rotational speed of the stepper motor could be controlled by sending different instructions, and the speed of needle insertion and withdrawal could be adjusted to meet various puncture needs. The calibration plate was used to install the calibration block with a specific size and pattern, and the camera of the Remebot could realize the calibration and registration of the tool centre point at the end of the puncture through identification of the calibration block.

### Model and radioactive seeds

A fresh pork specimen measuring 7 cm × 4 cm × 3 cm was uniformly encased in ultralight clay. Cavities, including the pterygopalatine fossa and posterior nasal aperture, were initially filled with sufficient ultralight clay. This approach ensured the stability of the tumour model, ensuring that it was securely established. The ultralight clay encasing the fresh pork was situated between the skull base and the bilateral mandibular ramus to simulate a skull base tumour. Holes were drilled using a dental handpiece from the left to the right mandibular angle along the inferior mandibular margin on the 1:1 skull model, with an interval of approximately 2 cm separating each pair of holes. Two holes were drilled bilaterally behind the hypoglossal canal and adjacent to the foramen magnum on the occipital bone of the skull model. An arch-shaped specimen of fresh pigskin measuring 15 cm × 10 cm was trimmed, and subcutaneous fat was removed to leave only the epidermis and dermis. The prepared fresh pigskin was affixed beneath the mandible and near the foramen magnum of the occipital bone using 3–0 sutures. The Kirschner wire was cut into 5-mm-long pieces for use instead of radioactive seeds to avoid radiation exposure to the researchers.

### Preoperative design

MK06A markers were affixed to the forehead and bilateral temporal regions of each skull model. The skull models were placed in a supine position for computed tomography (CT) imaging. The CT data were imported into Remebot 4.0 software for reconstruction and design of a radioactive particle implantation plan. The implantation needle trajectories and seed positions were planned for even distribution within the tumour model. A horizontal particle interval of 10 mm was set on the same axial plane of the CT images. Either two or three particles were implanted in each needle track. The deepest particle in each needle track was designated the target point. After retracting 10 mm from the target point along the needle track towards the surface, the position of the second particle was designed. In each needle track, 2 or 3 particles were implanted, for a total of 30 particles per skull model.

### Procedure

**Fig. 1:**
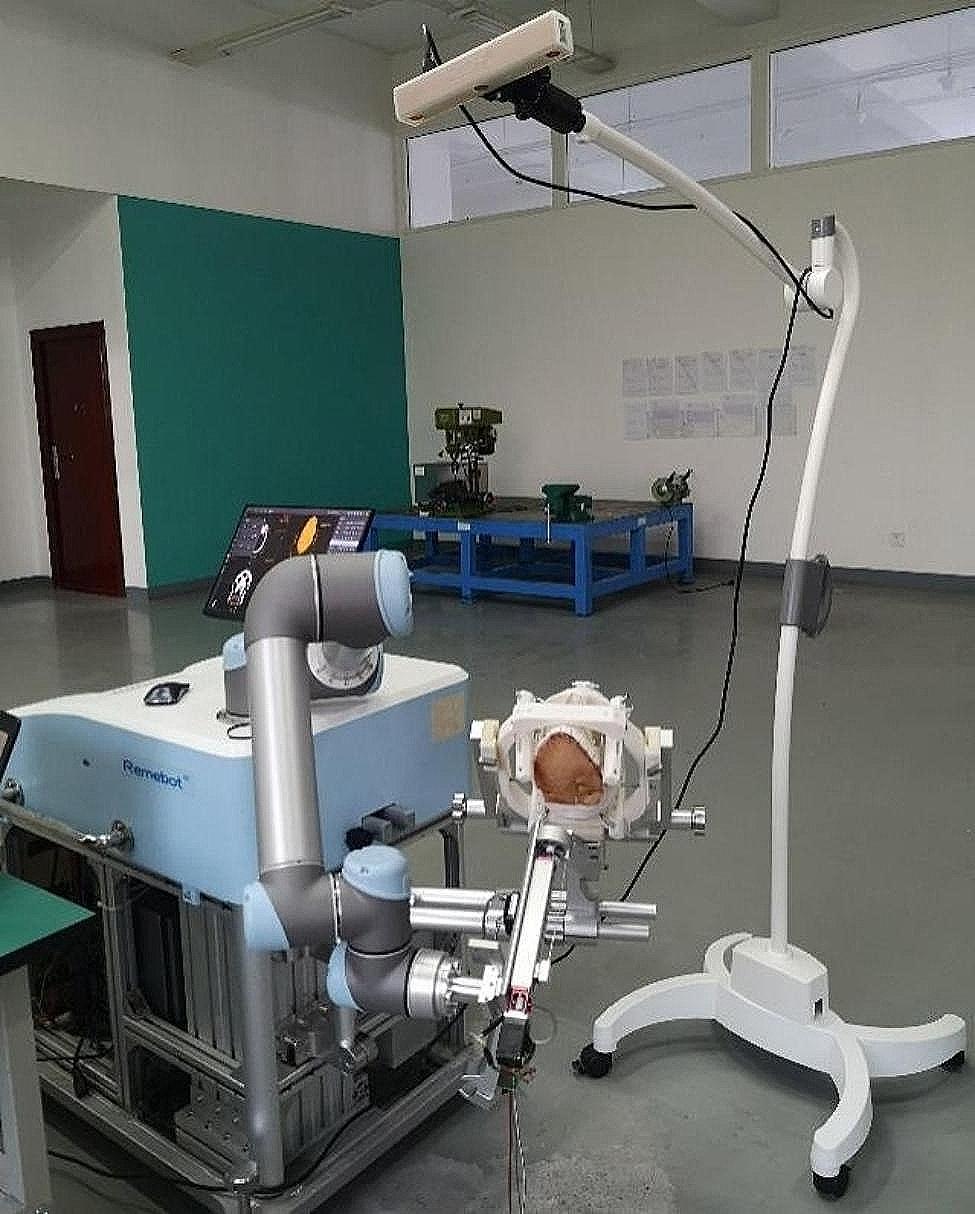
Following the stabilization of the skull model’s spatial position using a head frame, robot registration is performed

Following stabilization of the skull model’s spatial position using a head frame, robot registration was performed. (Fig. 1) To enable the automatic particle implantation device to perform the puncture action, the distance between the tip of the needle on the robotic arm’s puncture sheath and the target point was set to 70 mm. Once the robotic arm was in position, it was controlled via Remebot 4.0 software to advance axially 70 mm towards the target point, after which the skin was penetrated to reach the target site. Commands are input to the automatic implantation device, which advanced or retracted the inner needle to execute the particle implantation process. The robotic arm was manoeuvred to axially retract by 10 mm for the implantation of a subsequent particle, followed by an axial retraction of 60 mm to remove the sheath needle from the skin. Once the skin had resettled into its initial position, the spatial location of the actual skin puncture was marked using a navigation probe, and the corresponding spatial coordinates were noted within the software. The same technique was then applied for puncturing the following target point.

### Postoperative evaluation

After all the particles were implanted, a CT scan was performed again. The number of particles on the postoperative CT images was calculated to confirm whether the preoperative design was met for 30 particles. The data were imported into Remebot 4.0 software for reconstruction. The postoperative images were merged with the preoperative surgical plan. The pre- and post-surgical coordinates are denoted as (X1, Y1, Z1) and (X2, Y2, Z2), respectively.

$$d=\sqrt{\varDelta {X}^{2}+\varDelta {\text{Y}}^{2}+\varDelta {Z}^{2}}$$

The difference between the pre- and post-operative spatial coordinates of the radioactive particles was calculated to evaluate the accuracy of the fully automatic radioactive seed placement robot.

### Statistical analysis

Analysis was performed using IBM SPSS software version 20 (IBM Corp., Armonk, NY, USA). The placement error was calculated by comparing the preoperatively planned and actual postoperative placement points according to the Euclidean distance between the centres of the planned and actual placement points. One-way ANOVA was used for pairwise comparison of the placement error of the 30 particles implanted in the 4 skull phantom experiments, and a *p* value less than 0.05 was considered to indicate statistical significance.

## Results

**Fig. 2:**
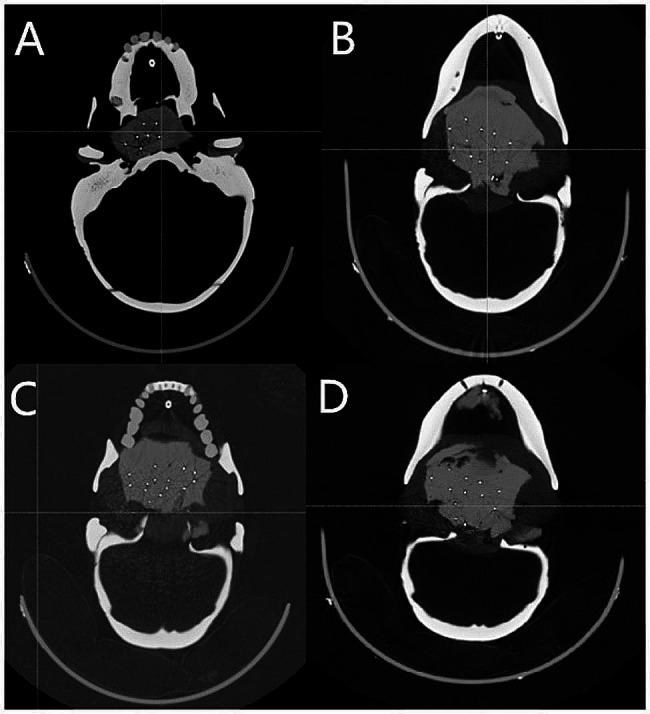
Postoperative CT images of 4 phantoms

**Fig. 3:**
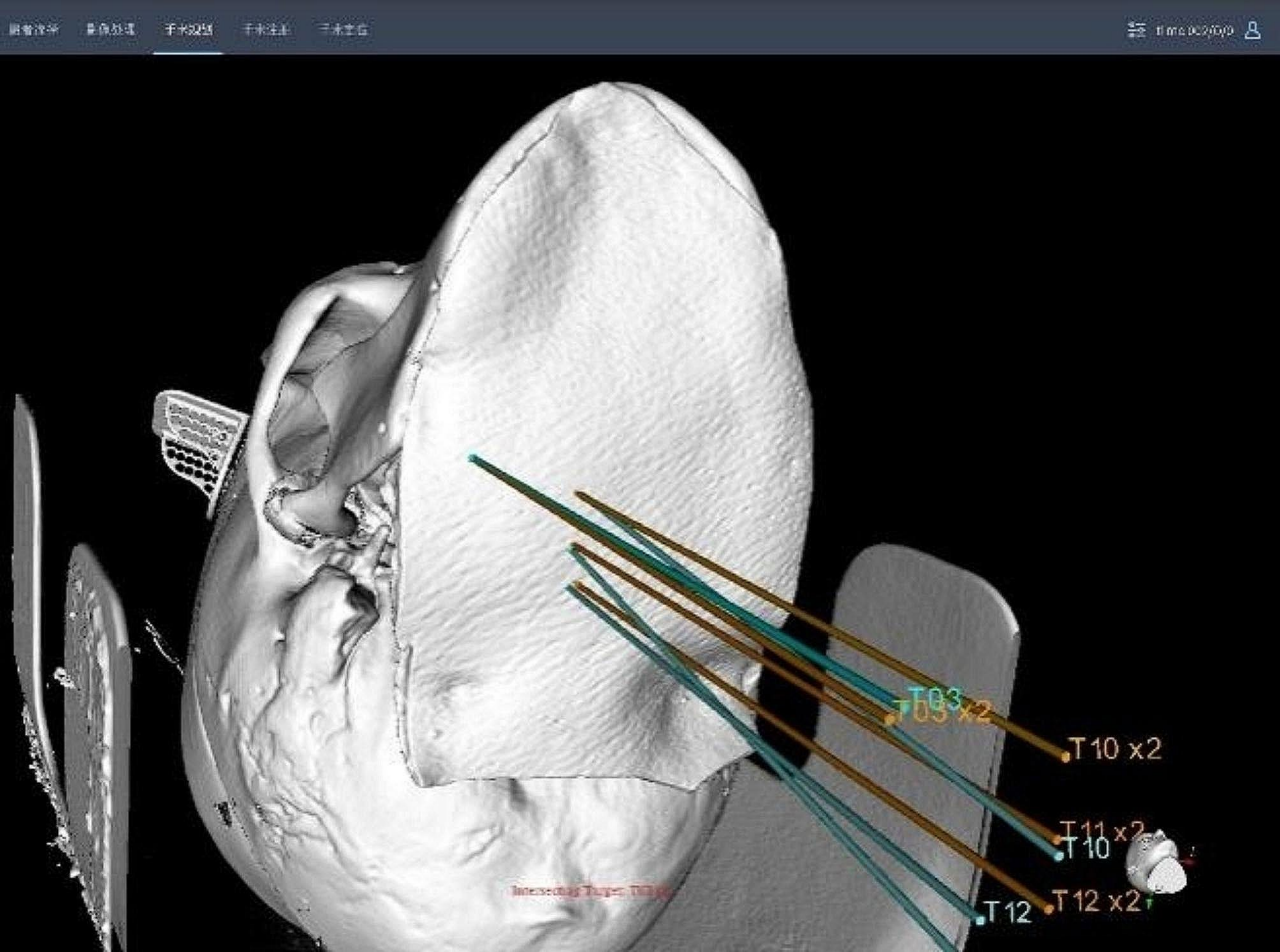
The superimposition of pre-(yellow) and post-(blue) needle pathway shows the deviation of puncture points and angle

A total of 120 seeds were implanted in 4 phantoms. The average error of seed placement positions was (2.51 ± 1.44) mm (Table 1; Fig. 2), Fig. 3 shows the result of superimposition of pre-(yellow) and post-(blue) needle pathway.

**Table 1:** One-way analysis of variance of phantom

Group	Mean ± SD(mm)	95% CI	p value
Phantom			0.195
Phantom 1	2.78 ± 1.75	2.12 ~ 3.43	
Phantom 2	2.34 ± 1.12	1.91 ~ 2.80	
Phantom 3	2.13 ± 1.04	1.74 ~ 2.51	
Phantom 4	2.80 ± 1.64	2.18 ~ 3.41	

## Discussion

Compared with external beam radiotherapy (EBRT), brachytherapy offers several advantages: (1) A high cumulative dose can be delivered to the target region, and the implantation of iodine-125 particles can maximally increase the dose delivered to the targeted area while significantly reducing the dose delivered to the surrounding normal tissues. (2) Compared with the fractionated, high-dose exposures involved in EBRT, brachytherapy consists of sustained, low-dose irradiation within the tumour, continuously targeting actively proliferating cells. (3) Radioactive seed implantation is highly conformal, minimizing trauma to normal tissues. Iodine-125 particle sources are characterized by a rapid decrease in γ-ray energy with increasing distance from the radiation source. (4) Following tumour reduction through treatment, particles tend to accumulate at the tumour’s centre, a phenomenon that maintains the radiation dose delivered to the target area to a certain degree as the radiation source decays [4]. Theoretically, the curative effect of brachytherapy highly depends on the accuracy of seed implantation. Poor accuracy may seriously affect the therapeutic efficacy and result in damage to the surrounding normal tissue. At present, surgeons can achieve accurate radioactive seed implantation with the assistance of 3D-printed templates [16, 17]. Nevertheless, surgeons still have to perform the procedure at the patient’s bedside. Thus, there still exists another inevitable issue, which is staff exposure, especially for surgeons with prolonged exposure to brachytherapy. If a robot could perform seed implantation in place of surgeons, the surgeons could stay at a safe distance from the radioactive seeds.

The last decade has witnessed major progress in the field of minimally invasive and robot-assisted surgeries [18], for instance, in skull base biopsy [19] and kidney [20] and breast [21] surgery. In prostate brachytherapy [22], a placement accuracy of millimetres is needed, and a needle placement accuracy of < 2.7 mm is generally considered acceptable in brachytherapy [23]. However, in the skull base, the precision requirement is greater, and robot-assisted needle insertion technology will help surgeons perform high-accuracy seed implantation. Currently, most related research has focused primarily on applications in treating liver [24], prostate [25], lung [26], and cervical [27] cancer, with few studies addressing the application of such robots in treating maxillofacial tumours. In 2017, Cui [28] established a surgical robot for radioactive seed implantation capable of automating radioactive seed placement. The robot’s mechanical structure design and the seed storage and propulsion device have been completed. The positional error for each robotic arm joint is less than 0.1 mm. In 2019, Zhu [29] reported the use of a fully automatic robot for radioactive particle implantation in treating skull base tumours. This technology is currently in the in vitro validation stage using models and cadaveric skulls, with an implantation accuracy of 1.41 ± 0.38 mm and 2.48 ± 0.32 mm, respectively. The surgical robots developed by Zhu and Cui both attach to either side of the operating table and can achieve movement with six degrees of freedom (DOF). The Remebot used in this study, however, is mounted on an operating trolley and can be moved freely within the operating room. It also features a 6-DOF mechanical arm that can precisely reach any target point and navigate paths within the surgical field, offering enhanced flexibility. As mentioned in several other studies on surgical robots, the sources of error of the Remebot mainly consist of the following components: the monitored accuracy, large orientation workspace, real-time tissue resistance monitoring, haptic control, maintenance program, sterilizable needle insertion device, CT scan compliance, automated safety control and fast manual control. Any component of the system can contribute to the overall systematic error. However, our preliminary work has verified the accuracy and safety of the Remebot device for performing skull base tumour punctures [30].

An eligible phantom could provide the possibility of overall quality assurance and specific quality control of the brachytherapy process. To simulate an authentic surgical scenario and mimic real tissue mechanics, we added fresh animal skin to the skull model to closely replicate the surgical puncture process. Previous research has reported that the total needle insertion force is the total of dissimilar forces spread sideways relative to the shaft of the insertion needle, for example, the cutting force, stiffness force, and frictional force [31–33]. Diverse types of needle tips may also affect skin deviation during puncture [34]. On the basis of our preliminary research and clinical experience, we found that placement error is significantly associated with deviations at the skin puncture point.

The results indicated that puncture errors varied across different skin areas. In the submandibular region near the mandible, that is, the outer edge of the skin, both the actual puncture point and the needle angulation exhibited minimal deviation. Conversely, larger deviations were observed in the central and posterior skin areas near the foramen magnum, which may be attributable to the varying skin tension in these areas. The skin at the outer edge was sutured to the inferior border of the mandible, resulting in greater tension and a reduced likelihood of shifting during puncture. In contrast, the more relaxed skin near the centre and foramen magnum was prone to deviation from puncture forces upon needle contact. Thus, the process of puncture needle insertion or retraction through the skin has an effect on accuracy. However, the mechanism of these needle-tissue interactions has not been elucidated. Consequently, predicting and compensating for needle deflection through calculations was not feasible, necessitating further research into the mechanisms of interaction between various needle tip shapes and skin.

The end effector for radioactive particle implantation currently under investigation is notably bulky [35]. The distance from the mechanical arm’s centre point to the puncture needle tip is approximately 31.6 mm, comprising a 10.8 mm puncture needle and a 20.8 mm device body. This design accommodates the need for needle insertion beneath the jaw or through the sigmoid notch of the mandible for most skull base tumours but necessitates a relatively long puncture trajectory. If entry is gained from beneath the jaw, the mechanical arm must manoeuvre the puncturing apparatus to the patient’s side. During the operation, there is a risk of colliding with the patient or failing to reach the intended position. Consequently, refining the end-effector to reduce its size and enhance its practicality is a necessary next step.

## Conclusion

This study presents an innovative, fully automated radioactive particle implantation system utilizing the Remebot device, which can successfully complete automated localization, needle insertion, and radioactive particle implantation procedures for skull base tumours. The phantom experiments showed the robotic system to be reliable, stable, efficient and safe. Currently, the precision of radioactive particle implantation robots can preliminarily meet the requirements of clinical surgery. Subsequent research will aim to further elucidate the mechanisms of interaction and deformational forces between the puncture needle and soft tissues.

## Data Availability

All data are calculated by the software itself. The datasets used and/or analyzed during the current study available from the corresponding author on reasonable request.
